# Chiari I Malformation and Spinal Cord Ischemia in a Cadaver

**DOI:** 10.7759/cureus.1567

**Published:** 2017-08-15

**Authors:** Faizullah Mashriqi, Marios Loukas, Rod J Oskouian, Anthony V D'Antoni, R. Shane Tubbs

**Affiliations:** 1 Department of Molecular, Cellular, and Biomedical Sciences, CUNY School of Medicine; 2 Department of Anatomical Sciences, St. George's University School of Medicine, Grenada, West Indies; 3 Swedish Neuroscience Institute; 4 Department of Molecular, Cellular and Biomedical Sciences, CUNY School of Medicine; 5 Neurosurgery, Seattle Science Foundation

**Keywords:** posterior fossa, herniation, hindbrain, tonsillar ectopia, neurosurgery, complications, injury

## Abstract

With advanced imaging, the Chiari I malformation (CIM) is more frequently diagnosed than in the past when this entity was identified most commonly at autopsy. Herein, we report the rare case of an adult cadaver found not only to have CIM but also adjacent spinal cord ischemia. This case is discussed in the context of chronic compression of the spinal cord by a CIM and the need for close monitoring of these patients.

## Introduction

Classically, Chiari malformations are a group of hindbrain herniations from the posterior-cranial fossa through the foramen magnum. The Chiari I malformation (CIM) is defined by caudal displacement of the cerebellar tonsils, usually greater than 3 mm below the plane of the foramen magnum. CIM is classically associated with multiple anatomic presentations, including anomalies of the craniovertebral junction, meninges, spinal cord, and ventricles. Craniovertebral junction anomalies are very common with an incidence of greater than 50%. These anomalies include underdevelopment of the occiput (which may result in a shortened posterior-cranial fossa), enlargement of the foramen magnum, and shortened clivus. Syringomyelia is the most common spinal lesion and an underlying syrinx can result in scoliosis [1]. Herein, we describe the cadaveric findings of a CIM and adjacent spinal cord ischemia.

## Case presentation

**Figure 1:**
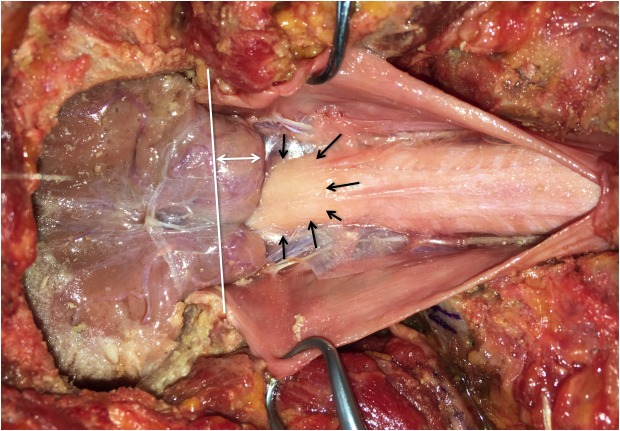
Posterior view of exposed craniocervical region with dura mater opened. The caudal descent of the cerebellar tonsils is evident as are the ischemic changes of the upper cervical spinal cord. The white line illustrates the plane of the foramen magnum; the white double arrows show the extent of herniation of the right-cerebellar tonsil below the foramen magnum. The black arrows outline the area of ischemic brainstem.

During the routine dissection of the craniocervical junction in a male cadaver who, at death, was 64 years old, the left and right cerebellar tonsils were found to lie 15 mm inferior to the foramen magnum and thus constituted a CIM (Figure 1). Additionally, the cervical spinal cord adjacent and below the herniated tonsils was seen to have signs of ischemia. Histological analysis of the ischemic cord found that the ischemia was chronic in nature and that there were no thromboses in the adjacent blood vessels, e.g., anterior or posterior spinal arteries. The ischemia was also more profound posteriorly, implying that the posterior vasculature had been subjected to more compression over time. No syringomyelia was found. Additionally, no signs of trauma or past operative intervention to the area were noted. The patient’s cause of death was a myocardial infarction. It is unknown if the patient had symptoms of either the CIM or spinal cord ischemia during life.

## Discussion

CIM can be either congenital or, more rarely, acquired later in life. There are many theories concerning the pathophysiology of CIM. Chiari attributed CIM to hydrocephalus, although we now know that less than 10% of CIM patients present with hydrocephalus [2-3]. Another theory has focused on mismatched rates of bone and brain development. Theoretically, a small posterior cranial fossa would limit proper brain development and might result in hindbrain herniation through the foramen magnum. Animal models with underdeveloped posterior cranial fossae support this theory [2]. The spinal cord tethering theory states that tension forces herniation of the hindbrain; however, this does not successfully explain other presentations of CIM [4]. The hydrodynamic theory is based on the craniospinal pressure gradient and dictates that pulsatile pressure from the supratentorial region causes cerebellar herniation and cerebrospinal fluid flow reversal, resulting in syringomyelia. The hydrodynamic theory does not properly explain acquired CIM (i.e., after multiple lumbar punctures) [2-3]. Stenosis of the subarachnoid space adjacent to the foramen magnum can cause CIM due to impaired cerebrospinal fluid flow [5]. This was demonstrated in patients with a post-pharyngitis pannus that resulted in CIM [2].

Patients with CIM typically present with pain that is localized to the occipital region; CIM should, therefore, be a differential etiology for occipital neuralgia, even though this presentation is rare [6]. Occipital pain is generally exacerbated by the Valsalva maneuver as explained by the hydrodynamic theory. Lower cranial nerve dysfunction can also be seen and may present as atrophy of the tongue, sleep apnea, nystagmus, and dysphagia with gagging [1].

Atypical presentations have been reported in the literature and include (1) Charcot arthropathy of the shoulder secondary to syringomyelia [7], (2) acute anterior spinal artery thrombosis with subsequent respiratory arrest and anoxic spinal and brain injury [8], and (3) a post-trauma cervical subdural hematoma complicated by the presence of a CIM [9]. The latter two cases are similar to the case reported herein as they demonstrate direct neural/vascular compression with subsequent injury. However, the association between CIM and spinal cord infarction (i.e., anterior spinal artery syndrome) is not a common finding [8]. The second case presented a 31-year-old female with a history of CIM but no cardiovascular morbidities. The patient presented with neck pain that radiated to both upper limbs. Ischemic infarction was seen on magnetic resonance imagining (MRI) in the cervical spine, and the magnetic resonance angiogram (MRA) revealed thrombosis of the anterior spinal artery. The patient suffered respiratory arrest and anoxic spinal injury. Although thrombosis of the anterior spinal artery was not seen in this case, one could consider possible long-term compression of the vessels supplying the upper spinal cord, such as the anterior-spinal artery, to be involved. 

## Conclusions

To our knowledge, this case represents the first report of a cadaver with CIM and obvious spinal cord ischemia. Although the cause of death in the reported cadaver was myocardial infarction, it is likely that the ischemic spinal cord was symptomatic, if not contributory, to the death. Such cases illustrate the need for careful monitoring of patients with CIM and to monitor those who have not had operative intervention even more closely. 
